# Gambling-like behavior in pigeons: ‘jackpot’ signals promote maladaptive risky choice

**DOI:** 10.1038/s41598-017-06641-x

**Published:** 2017-07-26

**Authors:** Aaron P. Smith, Joshua S. Beckmann, Thomas R. Zentall

**Affiliations:** Department of Psychology, University of Kentucky, Lexington, Kentucky 40506 United States of America

## Abstract

Individuals often face choices that have uncertain outcomes and have important consequences. As a model of this environment, laboratory experiments often offer a choice between an uncertain, large reward that varies in its probability of delivery against a certain but smaller reward as a measure of an individual’s risk aversion. An important factor generally lacking from these procedures are gambling related cues that may moderate risk preferences. The present experiment offered pigeons choices between unreliable and certain rewards but, for the Signaled group on winning choices, presented a ‘jackpot’ signal prior to reward delivery. The Unsignaled group received an ambiguous stimulus not informative of choice outcomes. For the Signaled group, presenting win signals effectively blocked value discounting for the large, uncertain outcome as the probability of a loss increased, whereas the Unsignaled group showed regular preference changes similar to previous research lacking gambling related cues. These maladaptive choices were further shown to be unaffected by more salient loss signals and resistant to response cost increases. The results suggest an important role of an individual’s sensitivity to outcome-correlated cues in influencing risky choices that may moderate gambling behaviors in humans, particularly in casino and other gambling-specific environments.

## Introduction

Individuals are often faced with choices involving uncertain outcomes that can have critical consequences such as predation in the wild or large financial losses. In the laboratory, risky environments are often modeled by offering a choice between an uncertain large reward (UL) against a certain but smaller reward (CS), where the odds against receiving the UL reward are systematically increased to determine how the value of the UL changes. Under this probability discounting (PD) procedure, the rate at which individuals discount the value of the UL with increased odds against its receipt^1^ indexes their risk tolerance as a measure of their propensity to take future risks^2^. Indeed, individual differences in risk tolerance are an important factor in risky decision making as these measures have shown clinical relevance through associations with gambling^3–5^, smoking^6, 7^, and internet gaming^8^ behaviors, as well as obesity^9^. Additionally, as the DSM-V has categorized gambling as an addictive disorder^10^, and a high prevalence of negative outcomes (monetary or otherwise) are associated with it^11–13^, determining the underlying processes involved in risky decision making may aid in understanding these maladaptive behaviors.

In a PD procedure, optimal decision makers should maximize their expected reward as described by normative theories such as expected value^14–16^ and optimal foraging theory^17^. Evidence suggests, however, that optimal choice does not always occur (e.g., refs 14 and 15). In PD experiments, choice often appears hyperbolic and is well described by Equation 1:

1 $$UL=\frac{A}{(1+h\,{\Theta })}$$

in which the value of the UL initially begins at *A* but is devalued as a function of the odds against [p/(1 − p)] receiving it (*Θ*) multiplied by *h*, a free parameter that reflects the degree of UL value discounting.

The rate at which individuals will discount the probability of an outcome has been shown to be influenced by factors such as the magnitude of the reward^18, 19^, the manner in which the probabilities are presented^20^, and asymmetries between decisions of gains and losses^21, 22^. Another potential but less explored factor is the influence of gambling related cues^23^. Two animal models of risky choice have shown cues to be efficacious moderators of risky decisions. For example, using a rodent version of the Iowa Gambling Task, Barrus and Winstanley^24^ found that the addition of audio-visual cues simultaneously presented with winning outcomes biased choice towards a UL-like alternative that provided less reinforcement overall relative to a condition without cues.

Another procedure^25^ used with pigeons and starlings found that signaling the outcome *before* actual receipt of the reward greatly promoted gambling-like choices^26–28^ that provided as little as 10% percent of the reward of the non-gambling alternative^29–31^. In this suboptimal choice procedure, choice of a gambling-like alternative is followed either by a signal indicating that a win or loss will follow, while the alternative choice generally results in an ambiguous, uninformative cue that provides greater overall reward^26^. It has been hypothesized that under these conditions pigeons over-weight the infrequent signal for wins^28, 29^, and show more optimal preferences if these signals are ambiguous^32, 33^; conversely, pigeons also appear to under-weight the signal for losses^30, 31, 34, 35^, and show little change in choice when the salience of the loss is manipulated^30, 35^. Similar effects involving signaled outcomes may also be relevant to human risk taking, as Molet *et al*.^36^ found using an analogous procedure. Specifically, individuals who engaged in commercial gambling behaviors chose the suboptimal gambling-like alternative significantly more than those who did not.

Although various mechanisms have been proposed for why the signals for wins promote suboptimal decision making to such an extent^27–29, 31^, the effect is robust and has yet to be employed for a variety of risky choice procedures such as PD. The suboptimal choice and PD procedures are similar, but there are two notable differences. First, strong suboptimal preferences have generally been found when the gambling-like alternative is compared to an alternative with an ambiguous signal not predictive of the outcome^27, 28^. In PD, the UL is often compared to the CS, or the certain small alternative that has no uncertainty as to its outcome. When similar conditions that lack uncertainty are used with suboptimal choice procedures, indifference or relatively weaker preferences are often found with 10 s signal durations^26, 27, 37–39^. Second, PD procedures usually employ magnitude discriminations of either differential amounts of money for humans^19^ or food rewards for animals^40, 41^. Many current theories as to why suboptimal choice occurs, however, are based upon procedures that primarily assess one dimension^29, 42^ of how the signals could be operating: its predictive utility for the presence or absence of the forthcoming reward^26–28, 31^. Furthermore, while procedures have parametrically assessed preferences for a predictive ‘jackpot’ signal against an ambiguous signal across a range of probabilities^28, 29, 31^ and differential magnitudes at single choice probabilities^43^, the interaction of the two has not been studied.

**Figure 1:**
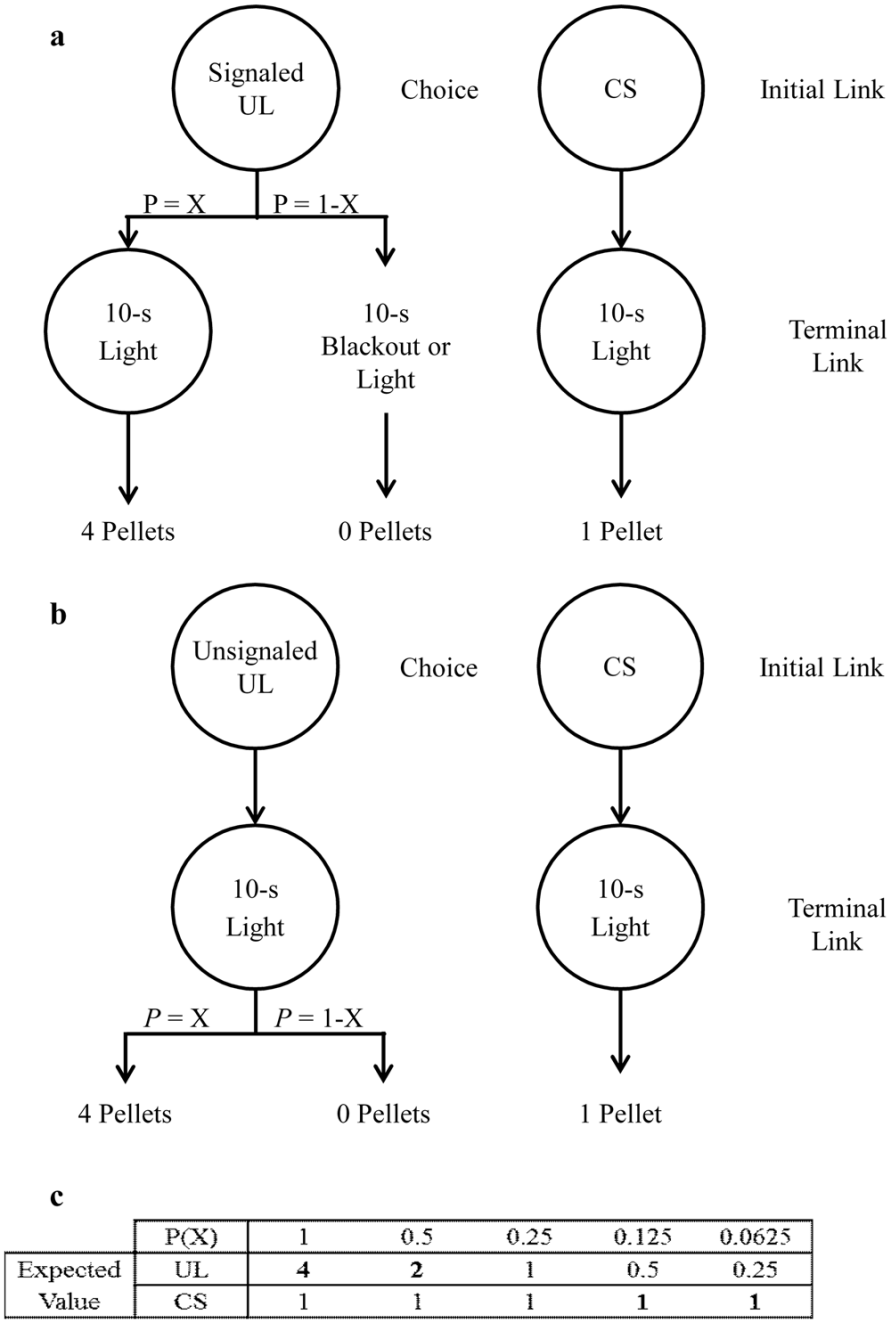
(**a**) Schematic of the general method for the Signaled group. (**b**) Schematic of the general method for the Unsignaled group. (**c**) Table indicating the probabilities of reinforcement used in determining UL reward delivery as well as the expected values for the UL and CS. *Note*: *bold numbers indicate the optimal choice*.

Therefore, the purpose of the present experiments was to extend the suboptimal choice research by testing how differential magnitudes of reinforcement interact with probabilistic outcomes within the framework of PD. To assess the effects of the signaled outcomes, the probability of receiving the UL reward (4 pellets) was gradually decreased over blocks of trials from 100% to 6.25% against a CS choice of a certain 1 pellet (see Fig. 1). For the Unsignaled group, choice of the UL always resulted in a stimulus that was not correlated with the outcome; this group served as an analogous control condition for procedures in which no differential cues are used^40, 41^. For the Signaled group, however, choice of the UL only resulted in a stimulus when the outcome was a win, or a ‘jackpot’ signal. If differential magnitudes of reinforcement, rather than predictive utility of the UL and CS signals, can produce a suboptimal choice effect, then the pigeons in the Signaled group should discount the UL less (smaller *h* values) than the Unsignaled group; however, if the suboptimal choice effect requires that the UL win signal has greater predictive utility for reward than the CS, the Signaled and Unsignaled groups should discount at similar rates.

## Results

**Figure 2:**
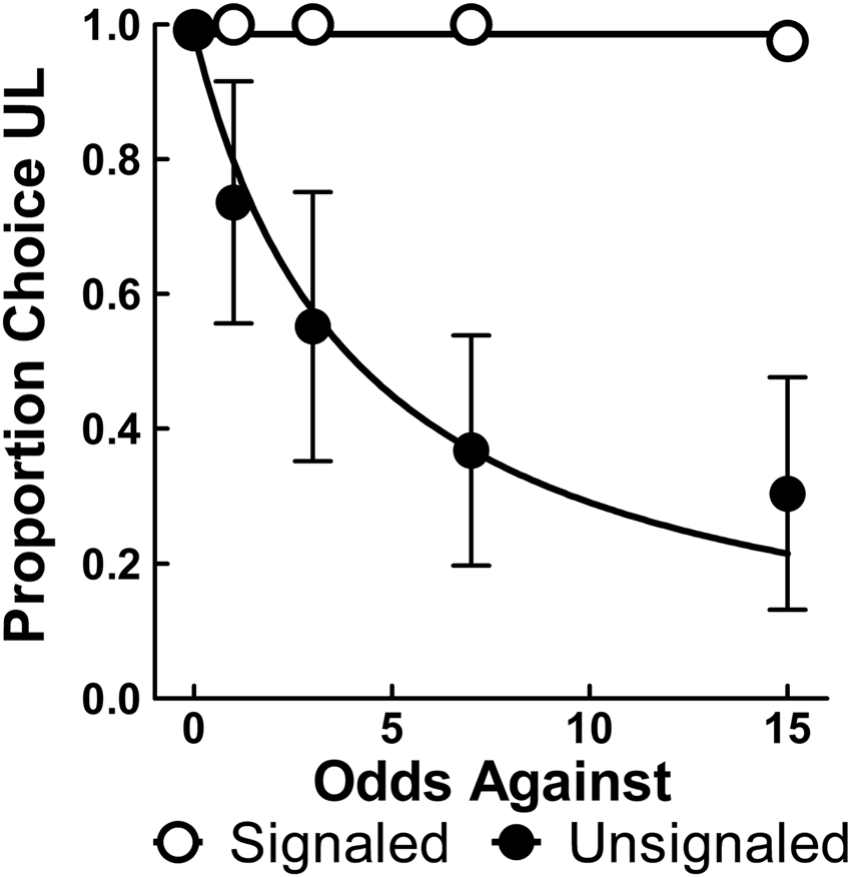
Mean (±SEM) proportion choice of the UL alternative averaged over the last five sessions of baseline training (*n* = 10). Slope parameter estimates (*h*) from Equation 1 were 0.24 for the Unsignaled group and <0.01 for the Signaled group.

Figure 2 shows the proportion of UL choices as a function of the odds against obtaining the UL reward averaged over the last five sessions of training while Supplementary Fig. S1 shows individual fits for all conditions. The Unsignaled group showed decreased choice of the UL as the reward rate began to favor the CS alternative (see Fig. 1c), indicative of sensitivity to the changes in primary reinforcement. As an index of these changes, the Unsignaled group crossed 0.5 proportion UL choices at a level suggesting one certain pellet was approximately equal in value to a 20% chance at four pellets. An indifference point of 20% is very similar to the point of equivalent expected values at 25% reinforcement (see Fig. 1c), suggesting the Unsignaled group nearly optimaly tracked changes in the rate of primary reinforcement. The Signaled group, however, showed no apparent change in choice, even when the reinforcement rates heavily favored the CS. Indeed, a non-linear mixed effects (nlme) analysis using a shared *A* parameter (*A* = 0.99, see Methods below) confirmed significant differences in discounting rates between the Signaled (*h* < 0.01, *SEM* = 0.01) and Unsignaled (*h* = 0.24, *SEM* = 0.07) groups, *F*(1, 38) = 12.98, *p* = 0.001, and further indicated that the *h* parameter for the Signaled group was not significantly different from zero, *p* > 0.999. These results effectively show that discounting of the UL outcome, while similar in appearance to previous studies with the Unsignaled group (e.g., refs 40 and 41), was completely blocked in the Signaled group.

### Reversal

A potential limitation of the reduced discounting is the use of a visual/spatial discrimination and presenting probabilities of reinforcement in a single decreasing order. Spatial discriminations of choices can confound spatial preferences (a pre-experimental preference for the left or right alternative) with a choice alternative preference^28, 37, 38^. Additionally, with similar procedures, the order of probabilities has been shown to alter preferences^44^. To address these issues, we used the same procedures as above but reversed the contingencies such that the UL alternative was now presented in the opposite location and the previous Signaled group became the Unsignaled group (see supplemental materials). If the large differences in preferences were indeed a product of the signal for wins and not a procedural artifact, the pigeons previously in the Unsignaled condition should now show attenuated discounting.

**Figure 3:**
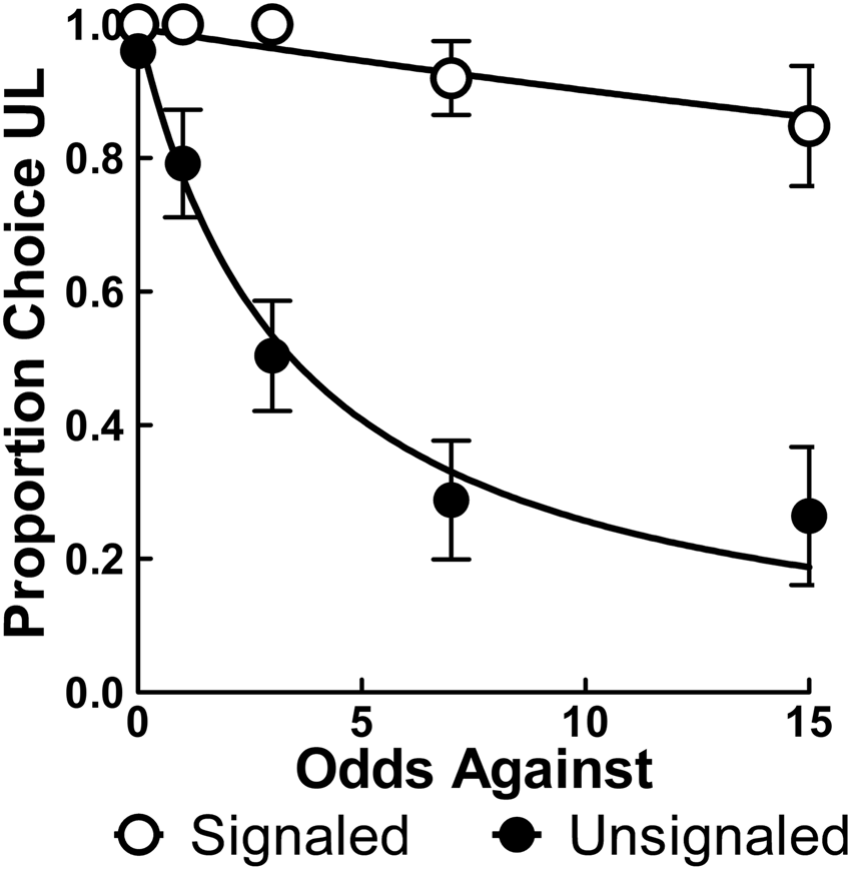
Mean (±SEM) proportion choice of the UL alternative averaged over the last five sessions of training following the reversal (*n* = 10). Slope parameter estimates (*h*) from Equation 1 were 0.29 for the Unsignaled group and 0.01 for the Signaled group.

Figure 3 shows the proportion of UL choice as a function of the odds against obtaining its reward averaged over the last five sessions of training while Supplementary Fig. S1 shows individual fits. The Signaled group, which previously discounted the UL, now showed minimal changes in UL preference as the odds against its delivery decreased (see Fig. 1c), while the Unsignaled group that previously did not discount the UL showed a large preference reversal. Additionally, the Unsignaled group’s indifference point (where UL preference crosses 0.5) was at 25% probability of reinforcement; this indicates that one certain pellet was approximately equal in value to a 25% chance of 4 pellets, the exact point at which the expected values become equivalent across the alternatives (see Fig. 1c). These trends were also confirmed by the nlme analysis with a shared *A* parameter (*A* = 1, see Methods below), in which the Unsignaled group (*h* = 0.29, *SEM* = 0.05) had a significantly steeper slope than the Signaled group (*h* = 0.01, *SEM* = 0.01), *F*(1, 38) = 37.28, *p* < 0.001. Furthermore, the slope parameter for the Signaled group was also not significantly different from zero, *p* = 0.201, again indicating a lack of discounting of the UL alternative’s value for the signaled group.

### Is it Suboptimal

**Figure 4:**
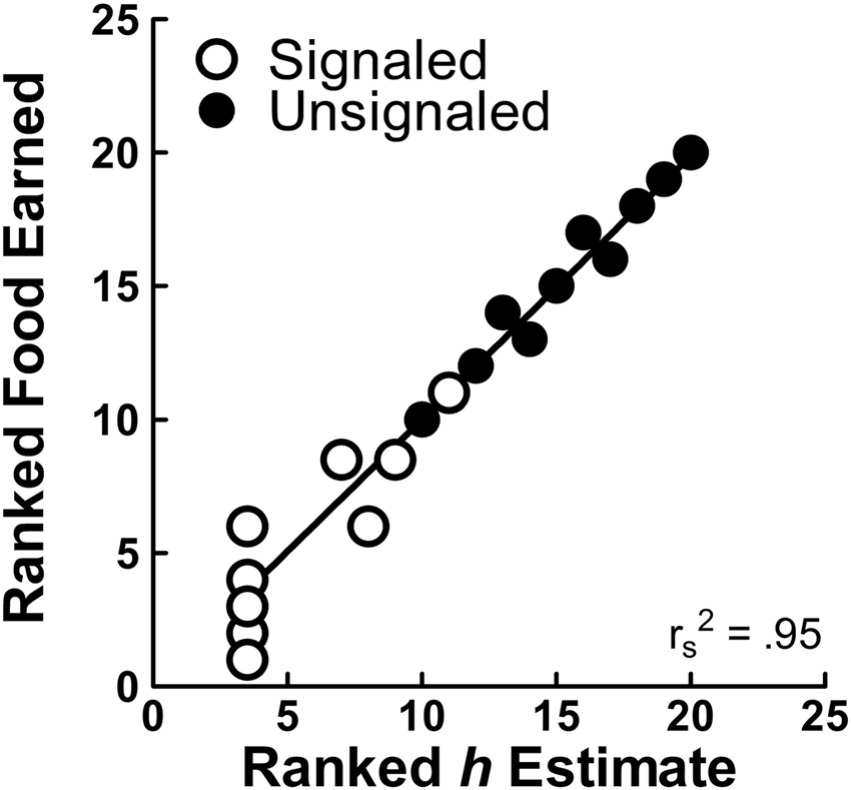
Spearman correlation of the cumulatively ranked obtained rewards for all birds in the signaled and unsignaled conditions as a function of ranked discounting estimates (parameter *h*; *n* = 20).

Animals are thought to have been pressured by their environments to behave optimally in order to survive^17^ and as such, they should prefer choice alternatives that produce higher probabilities of primary reinforcement^45^. This was the case in the Unsignaled conditions, where pigeons showed preference changes and indifference points that generally followed the scheduled reinforcement rates and tracked their relative expected value (see Fig. 1c). To confirm that scheduled reinforcement associated with the UL was actually less than optimal, in Table 1 we examined the obtained rewards for all birds in both conditions over the last five sessions on choice trials and found that obtained reinforcement was reduced. Pigeons’ preference for the UL outcome in the signaled conditions produced just over half (*M* = 51.2, *SEM* = 1.79) the reward earned in the Unsignaled conditions (*M* = 91.5, *SEM* = 6.12), clearly exemplifying suboptimal choice for the signaled conditions. Furthermore, Fig. 4 illustrates a positive correlation between discounting rates and obtained food rewards for all birds under both conditions, *r* ^*2*^ = 0.95, *p* < 0.001, suggesting that discounting within the range observed here advantageously led to increased reward.

**Table 1:** Cumulative obtained food over the last five sessions of training in the signaled and unsignaled conditions on free choice trials as a function of subject.

Subject		703	715	1016	1053	1870	2	707	1886	19845	22748	avg
**Condition**	**Signaled**	52	64	52	54	47	50	44	52	54	43	51.11
	**Unsignaled**	70	124	57	103	98	116	97	79	84	87	91.5

### Explicit Signaling of Losses

In the previous phases of this experiment there was a signal for winning outcomes but no signal for losses. Although there is a growing body of evidence to suggest that losses minimally influence preferences by pigeons^30, 34, 35^, translational procedures with rats have suggested that, if there is inhibition to loss signals, suboptimal choice does not occur^46^, and humans may show differential discounting of wins and losses^21, 22^. Although the lack of the ‘jackpot’ signal appearing on loss trials likely serves as a signal for a loss, we introduced a more salient signal for loss outcomes in the Signaled group to determine if salient losses would influence discount rates (see supplemental materials).

**Figure 5:**
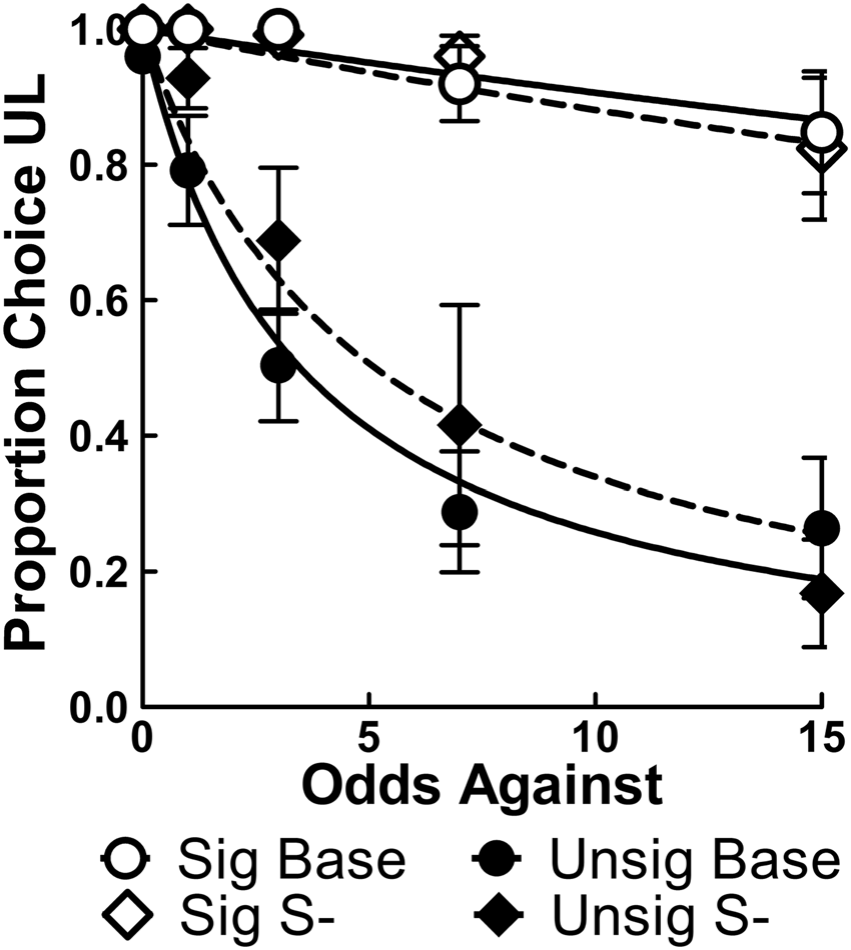
Mean (±SEM) proportion choice of the UL alternative averaged over the last five sessions of training from the reversal (circles and solid lines) and addition of the S- (diamonds and dashed lines) phases (*n* = 10).

Figure 5 shows the proportion of UL choices including the novel signaled losses (dashed lines) as well as the proportion of UL choices from the reversal (solid lines) for comparison. Despite losses now being cued, the Signaled group did not show any apparent change in UL preference, while the Unsignaled group showed a slight decrease in discounting. Nlme analysis using a shared *A* parameter (*A* = 1, see Methods below) revealed no significant changes after introducing a loss on *h* parameters for either the Signaled (*h* = 0.01, *SEM* = 0.01) or Unsignaled (*h* = 0.18, *SEM* = 0.04) group, *ps* ≥ 0.136, indicating the signal for losses had no effect on discounting. The Signaled group continued, however, to discount at a significantly reduced rate relative to the Unsignaled group, *F*(1, 86) = 54.51, *p* < 0.001, and discounting was not significantly different from zero, *p* = 0.136.

### Increasing the Cost to Gamble

As signaling losses did not reduce the Signaled group’s preference for the UL, we next asked if there were conditions under which the UL can be devalued for the Signaled group. Previous research has shown that altering the delay to reinforcement such that the UL has a longer delay relative to the CS^28^, decreasing the duration of the win signal prior to reinforcement^27, 28^, and decreasing the salience of the win signal^31^ can all decrease the effectiveness of signaled win outcomes. An alternative method is to increase the ‘cost’ or effort required to choose the UL.

To assess the effect of changing the cost on choice, we systematically increased the number of pecks required to choose the UL from 1 to 2, 4, 8, and 16 across session blocks while the cost of the CS remained at one peck (see supplementary materials). If an alternative has greater relative value, as the signaled UL appears to in the present experiment, its preference should decrease at a relatively slower rate, often described as being less *elastic* ^47, 48^. We therefore predicted that the Signaled group would show less elastic preference for the UL with increasing cost, relative to the Unsignaled group.

**Figure 6:**
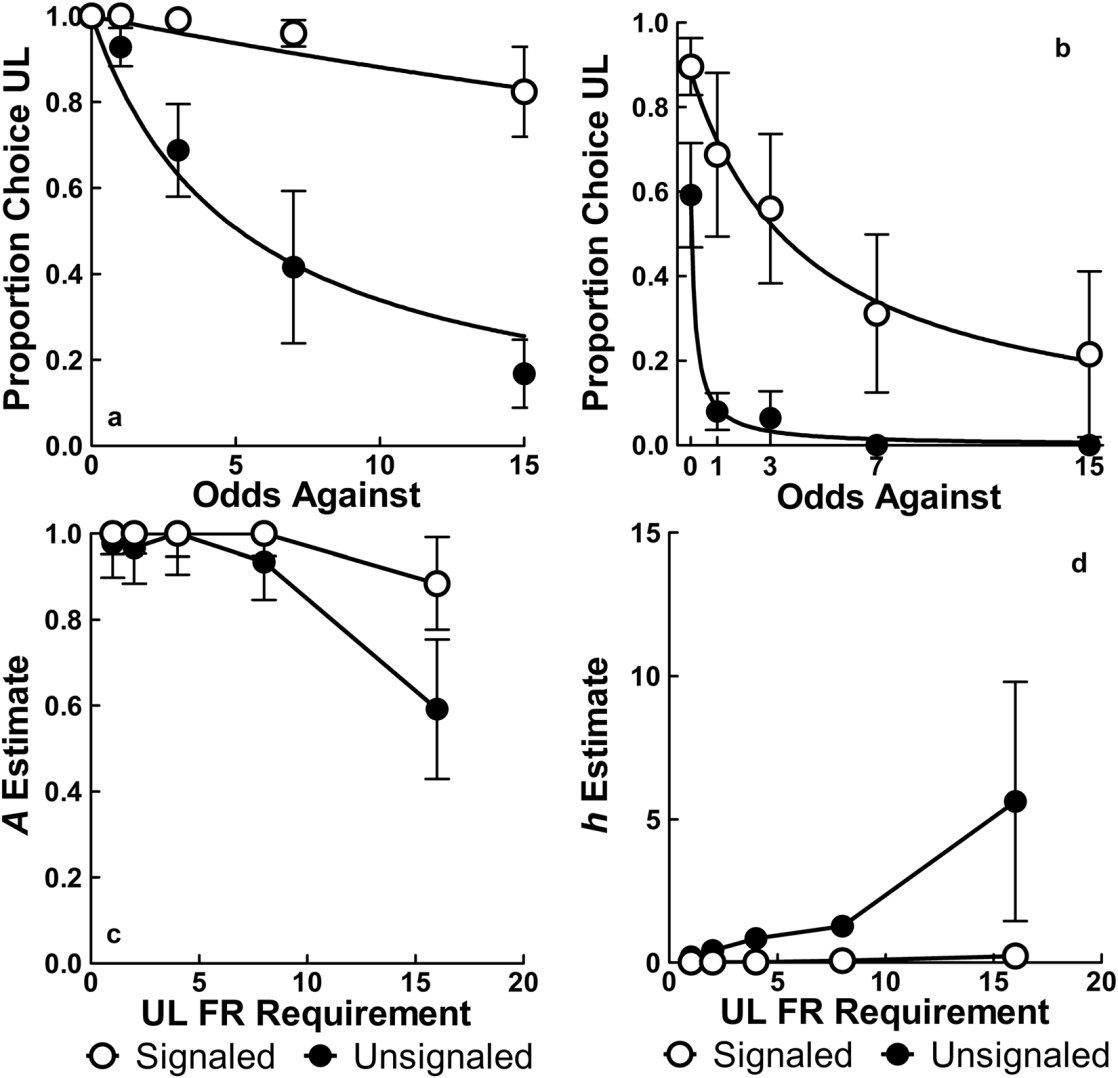
Top: mean (±SEM) proportion choice UL for the Signaled and Unsignaled groups at response costs of 1 (**a**) and 16 (**b**); additional costs can be seen in Supplementary Fig. S2. Bottom: mean (±SEM) *A* estimates (**c**) and *h* estimates (**d**) for the proportion choice UL (using Equation 1) as a function of UL response requirement (*n* = 10).

UL choice proportions at response costs of 1 and 16 as a function of the odds against receiving its reward are shown in the top row of Fig. 6; additional cost comparisons can be found in Supplementary Fig. S2. As the UL cost increased, both groups’ choice allocations showed increased discounting of the UL and a lowered intercept by the final cost of 16 responses, indicating that the increase in cost decreased the value of the UL alternative. The parameter estimates for *A* and *h* as a function of UL cost are also shown in the bottom row of Fig. 6 and illustrate these changes. Nlme analysis that included cost as an additional fixed factor and allowed the *A* parameter to vary for both groups also confirmed these effects as indicated by a significant Group × Cost interaction on discounting (*h* parameter), *F*(1, 233) = 14.24, *p* = 0.002, and main effects of group, *F*(1, 233) = 6.16, *p* = 0.0138, and cost, *F*(1, 233) = 31.01, *p* < 0.001, on the intercept (*A* parameter).

As predicted, the Unsignaled group showed a faster increase in discounting rates with increased cost. Both groups also showed decreased intercepts, indicating that when the cost was high enough, four pellets at 100% probability lost value relative to one pellet at a cost of one response. To better illustrate the changes in preference, the data were restructured as the average percent UL choice across the last five sessions of each UL peck requirement and fit with Equation 2 ^47, 48^:

2 $${\rm{l}}{\rm{o}}{\rm{g}}\,(\frac{UL}{UL+CS})={\rm{l}}{\rm{o}}{\rm{g}}\,({Q}_{0})+k\ast {ex}{{p}}^{((-\alpha {Q}_{0}x)-1)}$$

**Figure 7:**
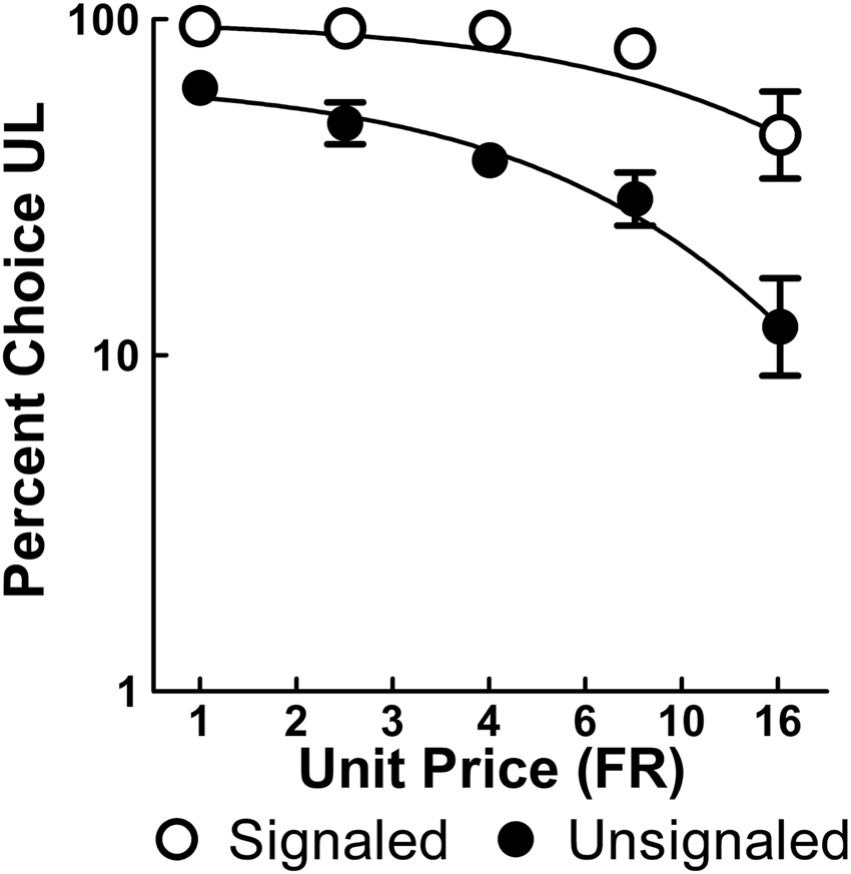
Mean (±SEM) percent choice of the UL alternative as a function of its response requirement fit with Equation 2 (*n* = 10).

In Equation 2, *Q*_*0*_ indicates the percent choice of the UL at the lowest cost (one response required), α indicates the rate at which UL preferences decreased (elasticity), and *k* is a scaling parameter. Illustrated in Fig. 7, the Signaled group initially chose the UL (averaging over odds against in blocks) to a greater extent (*Q*_0_ = 2.00, *SEM* = 0.03) than the Unsignaled group (*Q*_*0*_ = 1.82, *SEM* = 0.05) at the lowest cost of 1, *F*(1, 37) = 32.09, *p* < 0.001, consistent with the effects discussed above. Importantly, as the cost of UL choices increased, the Signaled group also showed less elasticity (α = 0.0056, *SEM* = 0.0024) than the Unsignaled group (α = 0.0148, *SEM* = 0.0035), by continuing to choose the UL despite the increase in cost, *F*(1, 233) = 6.87, *p* = 0.013. Collectively, the above analysis suggests that when wins were signaled, demand for the UL choice was greater than when that same choice was unsignaled.

## Discussion

Although components of the present results have been reported in previous experiments, the current work advances our understanding of suboptimal choice by collectively encompassing past and predicted results within one model. Similar to previous work^27–31, 43^, signaling uncertain choice outcomes prior to reward delivery greatly increased risk preferences. Previous research showing strong suboptimal preferences has generally occurred, however, when the predictive value of the signal following the UL is greater than the CS^26, 37^. In the present experiments, the predictive value between the UL and CS signals were equal, which can lead to indifference or relatively weaker preferences^37, 39^. With the addition of a magnitude difference, strong suboptimal choice was found even when the UL and CS signals were equally predictive.

Within the framework of PD, the interaction of an increased reward magnitude and predictive value of the UL ‘jackpot’ signal blocked discounting of the UL’s value which, we believe, is the first demonstration of such an effect in the literature. While pigeons and starlings have been previously shown to be insensitive to signaled probabilities of reinforcement^28, 29, 31, 38^ and suboptimal choice has been found with magnitude differences^43^, their combination had not been tested and led to the blocking of PD. The choice behavior of the Unsignaled group with uninformative signals is also in stark contrast to the Signaled group. The Unsignaled group served as a control for how risky choice tasks are often modeled without signals^41, 49^ and more optimally discounted the UL leading to nearly twice as much reward as the Signaled group. Reversing the conditions^26, 27^ and providing a more salient loss signal^30, 34, 35^ further revealed that the difference between the two groups was not due to procedural artifacts and is consistent with previous research. Finally, a novel finding was that when the cost of UL choices was increased, demand for the UL was found to be more inelastic for the Signaled group.

Why signaling win outcomes reduced loss aversion to such an extent, however, is still unclear. For example, we have interpreted the group effects as the ‘jackpot’ signal reducing the effect of discounting (as do current theories of suboptimal choice), but it may also be that presenting a probabilistic cue that does not produce food (as in the Unsignaled group) produces increased discounting. In either case, while the discounting Equation 1 is useful in characterizing differences between signaled and unsignaled conditions, it does not offer clear explanations for why the differences occur. Several variables influencing the effectiveness of the win cues have been previously identified^26, 27, 31^, such as its predictive utility for reward, the duration of its appearance prior to reward, and its overall conditioned reinforcement value, leading to different hypotheses. One hypothesis stems from the value of information provided by the win signal^30, 31^. That is, the appearance of a signal for reward reduces the time spent in uncertainty of the reward. In the present experiment, however, the signaled condition had equally informative cues between the UL and CS alternatives. The fact that suboptimal preferences still emerged may therefore challenge this interpretation.

Alternative hypotheses stem from the value of win signals as conditioned reinforcers^27–29^. The stimulus value hypothesis^29^, based on the contextual choice model^42^, posits that the multidimensional conditioned reinforcement strength^37^ of the win signal (magnitude, predictive utility, cost, etc.) drives suboptimal choice (see supplementary materials and Fig. S3). As the CS and UL had equally informative cues for reward in the signaled conditions, only the dimensions of relative probabilities and magnitudes of reinforcement were different. Given that the pigeons were insensitive to the probabilities of reinforcement; the stimulus value hypothesis suggests that group differences in the present experiment were due to an increased sensitivity to the reward magnitude of the UL relative to the CS. As the actual UL magnitude of reward between the signaled and unsignaled conditions on win trials was 4 pellets, however, it is instead inferred that the ‘jackpot’ cues in the signaled condition effectively acted by increasing the magnitude of the UL reward.

The hyperbolic decay model has also been applied to risky choice^28, 50^. Hyperbolic decay suggests that the value of a choice alternative is determined by its delay to reinforcement. For probabilistic choices, however, an alternative’s value only decays when a signal predicting reinforcement is present. The value of a signal is initially set to 1, decays the longer it is present without reinforcement, and sums across trials of non-reinforcement. For example, in the unsignaled conditions, the CS signal is always followed by reinforcement 10 s after it appears and equates to 10 s of devaluation. The UL signal, however, is only sometimes followed by food; this means the UL can appear for 10, 20, or 30 s (etc.) across multiple trials prior to reinforcement. Greater UL devaluation is therefore consistent with the UL discounting seen in the unsignaled conditions and predicts increased CS preference as the probability of UL reinforcement declines. For the signaled conditions, the CS signal is also always followed by reinforcement, but the UL signal only appears when reinforcement will follow. Thus, even across diminishing UL reinforcement probabilities, both the CS and UL signals are equally subjected to 10 s of devaluation and individuals should be indifferent between them. While individuals in the signaled condition were indeed unaffected by diminishing UL reinforcement probabilities, they showed a strong preference for the UL rather than being indifferent between the UL and CS. In order to account for the present findings, a small addition of a magnitude term would need to be added^50^. Upon doing so, the initial value for the UL and CS changes to 4 and 1, respectively. Thus, the hyperbolic decay model is consistent with the present findings and predicts the current group differences are due to the combined effects of a signal occurring only when reinforcement follows and the magnitude of the UL being greater than the CS. Additionally, it may be possible for the hyperbolic decay model to account for the cost manipulation conducted here by accounting for the increased time it takes to complete the response requirement^50^.

Finally, the contrast^26^ and signal for good news (SiGN)^27^ hypotheses suggest that it is the change from a state of uncertainty (when making a probabilistic choice) to a state of certainty (when the signaled win outcome appears) that produces the suboptimal choice effect. As the outcome of the CS in the present experiments can be predicted at the time of choice (a certain one pellet), this alternative produces no contrast and, in the case for the SiGN hypothesis, would not serve as a conditioned reinforcer. The UL, however, cannot be predicted at the time of choice and, upon the appearance of the ‘jackpot’ signal, generates contrast or an increase in reinforcement value that leads to suboptimal preference. In their present form, the contrast and SiGN hypotheses both would predict suboptimal preferences in the present experiment. The SiGN hypothesis also states that, because the UL win signal appears temporally closer to reinforcement than the CS choice stimulus, the appearance of the UL win signal reduces the delay to reinforcement. With this added component, the SiGN hypothesis has been able to account for changes in suboptimal preferences based on changing delays to reinforcement^51^ and the cost manipulation conducted here (as it increases the UL’s delay to reinforcement) that the contrast explanation currently cannot. Neither hypothesis, however, makes any assertion as to the role of differential magnitudes of reinforcement, although it follows to reason that signals predicting greater magnitudes of reinforcement could be conceptualized to produce greater contrast and/or reinforcement value than signals predicting smaller magnitudes. Still, the present results require that these models better formulate their predictions of how other dimensions of reinforcement may interact.

Although the present experiments cannot clearly distinguish between these different models, the results presented here better support hypotheses stemming from the reinforcing value of the ‘jackpot’ signal rather than its information. The general finding that ‘jackpot’ cues have following a risky choice is a robust phenomenon in animal models^24, 26, 31, 52–54^, implicating an important role of cues on an individual’s risky decision making. Laboratory measures of risk taking such in humans, however, do not often assess the role that cues may have on risk.

If laboratory measures such as PD are to inform other risky decisions such as gambling in humans^2^, these measures should also take into account the individual’s sensitivity to ‘jackpot’ signals. While evidence exists that human gamblers show increased physical arousal or gambling intentions^55, 56^ and regional fMRI brain activation to gambling-related scenarios or stimuli^57, 58^, fewer experiments have examined the role of outcome-correlated cues modulating gambling behavior^23^, although one study, using a reinforcement learning model, indicated that cues can effect choices when reinforcement rates were equivalent^59^.

Human gamblers have also shown reduced fMRI reward pathway activation to risky choice outcomes relative to healthy controls^60, 61^. This has led to the suggestion that, similar to substance abusers, gamblers seek highly rewarding events to compensate for a hypoactive reward system. Additionally, there is evidence that, relative to controls, gamblers show increased brain activity during anticipation of an expected win following risky choices^62^ and both humans and animals have shown increased neuronal activity during uncertainty prior to receiving a reward^63^. These findings suggest that the period after choice but prior to the outcome are an important factor in biasing risk preferences. Indeed, a procedure analogous to the signaled outcomes used here showed that individuals who are self-described gamblers increased choice of gambling-like alternatives^36^. These results suggest that outcome-correlated cues may indeed modulate human risk sensitivities relevant to certain behavior (e.g. gambling), but this needs to be verified in future research. Additionally, the effect of outcome-correlated cues may be different depending on whether they precede or occur simultaneously with the outcome, and future research should take this point into consideration.

The present experiments show that signaling a win prior to receipt of its outcome effectively increases risk taking and can block PD in pigeons. Furthermore, signaling losses do not attenuate the effect, and the value added by these signaled wins is resistant to increases in cost. Collectively, the results suggest that, when making risky decisions, stimuli correlated with win outcomes can increase risk to the point of suboptimality. Indeed, numerous examples of signaling stimuli prior to gambling outcomes occur in casinos, such as the images on the reels of a slot machine, the ball on a roulette wheel, and matching numbers on lottery and Powerball tickets. That pigeons in the Signaled group were also willing to pay an increased cost for the chance to obtain the ‘jackpot’ reward may also be an indicator of why some individuals can expend increasing resources gambling. Future gambling research and laboratory measures of an individual’s risk sensitivity should therefore assess the effect of such cues by controlling for their presence (and absence) to further determine their influence on decision-making.

## General Methods

Ten White Carneau pigeons approximately 8–12 years old originally purchased from the Palmetto Pigeon Plant (Sumter, SC) with previous experience in suboptimal choice tasks and no systematic differences in experience were used in the experiment. Subjects were housed in individual cages measuring 28 × 38 × 30.5 cm and maintained at 80–85% their free feeding weight on a 12:12 light-dark cycle (lights off at 7 pm) with free access to grit and water. All research was approved by the University of Kentucky Institutional Animal Care and Use committee (Protocol 01029L2006) and was conducted according to the 2010 *NIH Guide for the Care and Use of Laboratory Animals* (8^th^ edition).

The experiment was conducted in a Med Associates (St. Albans, VT) modular operant chamber (ENV-008) measuring 30.5 × 25.5 × 33 cm inside a noise attenuating box. The pigeons responded to three circular keys approximately 21.5 cm above the floor, 2.5 cm across, and 5 cm apart. A 12-stimulus inline projector (Industrial Electronics Engineering, Van Nuys, CA) behind each key projected one of four stimuli (red, green, or three white horizontal or vertical lines on a dark background) onto the left and right response keys and a white light onto the center key. Reinforcement was delivered to a magazine tray at the base of the response panel in the form of a 45-mg pellet from a dispenser (ENV-45 Med Associates, Fairfax, VT) behind the response keys. The chamber was illuminated by a 28 V, 0.1 A house light centered over the chamber. White noise was generated from outside the chamber and a computer in an adjacent room controlled the experiment using Med-PC IV.

Subjects were first trained using an autoshaping procedure in which one of four stimuli were illuminated randomly onto either the left or right response keys; the white light was only presented on the center response key. Following either 30 s or a peck to the stimulus, whichever came first, the house light illuminated and a single pellet delivered into the magazine. The house light remained illuminated for 5 s and then offset for 5 s resulting in a 10-s intertrial interval (ITI). This procedure for reinforcement and the ITI remained consistent throughout the experiment.

Following pretraining, subjects were trained on a visual/spatial one versus four pellet magnitude discrimination. All trials began with a white orienting stimulus on the center key. A response to the center key turned off the orienting stimulus and began either a forced or free choice trial. On free choice trials, concurrently available initial link stimuli of three horizontal or vertical white lines on a black background on each side key appeared. Choice of the uncertain large (UL) alternative led to a terminal link stimulus (red or green) for 10 s after which four pellets were delivered to the magazine. Choice of the certain small (CS) alternative led to a different terminal link stimulus (red or green) for 10 s after which a single pellet was delivered to the magazine. Forced choice trials were identical to free choice trials except that only one alternative appeared on either the left or right key. Sessions consisted of 65 trials, 25 free and 40 forced, divided into five 13-trial blocks. The first eight trials of each block were forced and the last five were free choice. All initial and terminal link stimuli (including their spatial location) were counterbalanced across subjects. Magnitude training continued until all subjects chose the UL alternative at least 95% of the time for two consecutive sessions

Subjects were then randomly assigned to the Signaled and Unsignaled groups and trained on a PD procedure structured similar to magnitude training. Each block began with eight forced trials followed by five free choice trials. The first block of trials of each session was the same as magnitude training. In subsequent blocks, the probability of receiving the UL reward when chosen decreased from 100% to 50%, 25%, 12.5%, and 6.25%. For the Signaled group, choice of the UL in these subsequent blocks led either to the predictive terminal link stimulus (or ‘jackpot’ signal) for 10 s followed by four pellets or a blackout period for 10 s. For the Unsignaled group, choice of the UL was always followed by the nonpredictive terminal link stimulus for 10 s that was followed by the four-pellet reward according to the probabilities of reinforcement associated with that block. Training continued until a line fit to the slope estimates (parameter *h*) was not statistically different from zero in both groups for five sessions, totaling 30 sessions.

### Data Analysis

Data were analyzed using nonlinear mixed effects (nlme) modeling using Equation 1 from the nlme package in R^64^. Estimates for both *A* and *h* parameters were generated treating group as a nominal factor and subject as a random factor. Two models were run that either allowed the *A* intercept parameter to vary for each group or as a global parameter shared by both groups. Model selection was chosen based on differences in the Akaike information criteria reaching at least 4 units lower^65^, (data not shown). As *h* estimates appeared non-linear in form, correlations including this measure used the ranked Spearman correlation.

### Data Availability

All data presented in the main document can be found as an online supplementary file.

## Electronic supplymentary material

Supplementary Information
Supplementary Dataset

## References

[1] Rachlin H, Raineri A, Cross D (1991). Subjective probability and delay. Journal of the Experimental Analysis of Behavior.

[2] Petry, N. M. & Madden, G. J. Discounting and pathological gambling (2010).

[3] Holt DD, Green L, Myerson J (2003). Is discounting impulsive?: Evidence from temporal and probability discounting in gambling and non-gambling college students. Behavioural processes.

[4] Petry NM (2012). Discounting of probabilistic rewards is associated with gambling abstinence in treatment-seeking pathological gamblers. Journal of abnormal psychology.

[5] Madden GJ, Petry NM, Johnson PS (2009). Pathological gamblers discount probabilistic rewards less steeply than matched controls. Experimental and clinical psychopharmacology.

[6] Reynolds B, Richards JB, Horn K, Karraker K (2004). Delay discounting and probability discounting as related to cigarette smoking status in adults. Behavioural processes.

[7] Yi R, Chase WD, Bickel WK (2007). Probability discounting among cigarette smokers and nonsmokers: molecular analysis discerns group differences. Behavioural pharmacology.

[8] Lin, X., Zhou, H., Dong, G. & Du, X. Impaired risk evaluation in people with Internet gaming disorder: fMRI evidence from a probability discounting task. *Progress in Neuro*-*Psychopharmacology and Biological Psychiatry* **56**, 142–148, doi:http://dx.doi.org/10.1016/j.pnpbp.2014.08.016 (2015). 10.1016/j.pnpbp.2014.08.016 25218095

[9] Rasmussen, E. B., Lawyer, S. R. & Reilly, W. Percent body fat is related to delay and probability discounting for food in humans. *Behavioural Processes* **83**, 23–30, doi:http://dx.doi.org/10.1016/j.beproc.2009.09.001 (2010). 10.1016/j.beproc.2009.09.001 19744547

[10] APA. *Diagnostic and statistical manual of mental disorders* (*5th ed*.) (Author, 2013).

[11] Hodgins DC, Stea JN, Grant JE (2011). Gambling disorders. The Lancet.

[12] Potenza MN, Fiellin DA, Heninger GR, Rounsaville BJ, Mazure CM (2002). Gambling. Journal of General Internal Medicine.

[13] Lesieur HR (1992). Compulsive gambling. Society.

[14] Herrnstein RJ (1990). Rational choice theory: Necessary but not sufficient. American Psychologist.

[15] Kanehman D, Tversky A (1979). Prospect theory: an analysis of decision under uncertainty. Econometrica.

[16] Starmer C (2000). Developments in non-expected utility theory: The hunt for a descriptive theory of choice under risk. Journal of economic literature.

[17] Stephens, D. W. & Krebs, J. R. *Foraging theory* (Princeton University Press, 1986).

[18] Green L, Myerson J, Ostaszewski P (1999). Amount of reward has opposite effects on the discounting of delayed and probabilistic outcomes. Journal of Experimental Psychology: Learning, Memory, and Cognition.

[19] Myerson J, Green L, Morris J (2011). Modeling the effect of reward amount on probability discounting. Journal of the Experimental Analysis of Behavior.

[20] Yi R, Bickel WK (2005). Representation of odds in terms of frequencies reduces probability discounting. The Psychological Record.

[21] Estle SJ, Green L, Myerson J, Holt DD (2006). Differential effects of amount on temporal and probability discounting of gains and losses. Memory & Cognition.

[22] Shead NW, Hodgins DC (2009). Probability discounting of gains and losses: Implications for risk attitudes and impulsivity. Journal of the experimental analysis of behavior.

[23] Barrus, M. M., Cherkasova, M. & Winstanley, C. A. In *Behavioral Neuroscience of Motivation* 507–529 (Springer, 2015). 10.1007/7854_2015_393 26531068

[24] Barrus MM, Winstanley CA (2016). Dopamine D3 receptors modulate the ability of win-paired cues to increase risky choice in a rat gambling task. The Journal of Neuroscience.

[25] Kendall SB (1974). Preference for intermittent reinforcement. Journal of the Experimental Analysis of Behavior.

[26] Zentall TR (2016). Resolving the paradox of suboptimal choice. Journal of Experimental Psychology: Animal Learning and Cognition.

[27] McDevitt MA, Dunn RM, Spetch ML, Ludvig EA (2016). When good news leads to bad choices. Journal of the Experimental Analysis of Behavior.

[28] Mazur JE (1996). Choice with certain and uncertain reinforcers in an adjusting-delay procedure. Journal of the experimental analysis of behavior.

[29] Smith AP, Bailey AR, Chow JJ, Beckmann JS, Zentall TR (2016). Suboptimal choice in pigeons: Stimulus value predicts choice over frequencies. PloS one.

[30] Fortes, I., Vasconcelos, M. & Machado, A. Testing the Boundaries of “Paradoxical” Predictions: Pigeons Do Disregard Bad News. *Journal of experimental psychology*. *Animal learning and cognition* (2016). 10.1037/xan0000114 27598063

[31] Vasconcelos M, Monteiro T, Kacelnik A (2015). Irrational choice and the value of information. Scientific Reports.

[32] Stagner J, Zentall T (2010). Suboptimal choice behavior by pigeons. Psychon Bull Rev.

[33] Spetch ML, Belke TW, Barnet RC, Dunn R, Pierce WD (1990). Suboptimal choice in a percentage-reinforcement procedure: Effects of signal condition and terminal-link length. Journal of the experimental analysis of behavior.

[34] Laude JR, Stagner JP, Zentall TR (2014). Suboptimal choice by pigeons may result from the diminishing effect of nonreinforcement. Journal of Experimental Psychology: Animal Learning and Cognition.

[35] Pisklak JM, McDevitt MA, Dunn RM, Spetch ML (2015). When good pigeons make bad decisions: Choice with probabilistic delays and outcomes. Journal of the Experimental Analysis of Behavior.

[36] Molet M (2012). Decision making by humans in a behavioral task: Do humans, like pigeons, show suboptimal choice?. Learning & Behavior.

[37] Smith, A. P. & Zentall, T. R. Suboptimal Choice in Pigeons: Choice Is Primarily Based on the Value of the Conditioned Reinforcer Rather Than Overall Reinforcement Rate. *Journal of Experimental Psychology*: *Animal Learning and Cognition* **42**, 212–220, doi:http://dx.doi.org/10.1037/xan0000092 (2016). 10.1037/xan0000092 26881902

[38] Zentall TR, Laude JR, Stagner J, Smith AP (2015). Suboptimal choice by pigeons: Evidence that the value of the conditioned reinforcer determines choice not the frequency. The Psychological Record.

[39] Stagner JP, Laude JR, Zentall TR (2012). Pigeons prefer discriminative stimuli independently of the overall probability of reinforcement and of the number of presentations of the conditioned reinforcer. Journal of Experimental Psychology: Animal Behavior Processes.

[40] Onge JRS, Abhari H, Floresco SB (2011). Dissociable contributions by prefrontal D1 and D2 receptors to risk-based decision making. The Journal of Neuroscience.

[41] Green L, Myerson J, Calvert AL (2010). Pigeons’ discounting of probabilistic and delayed reinforcers. Journal of the Experimental Analysis of Behavior.

[42] Grace RC (1994). A contextual model of concurrent-chains choice. Journal of the Experimental Analysis of Behavior.

[43] Zentall TR, Stagner J (2011). Maladaptive choice behaviour by pigeons: an animal analogue and possible mechanism for gambling (sub-optimal human decision-making behaviour). Proceedings of the Royal Society B: Biological Sciences.

[44] Yates, J. R. *et al*. Effects of NMDA receptor antagonists on probability discounting depend on the order of probability presentation. *Pharmacology Biochemistry and Behavior* **150–151**, 31–38, doi:http://dx.doi.org/10.1016/j.pbb.2016.09.004 (2016). 10.1016/j.pbb.2016.09.004 PMC5145760 27642050

[45] Bailey JT, Mazur JE (1990). Choice behavior in transition: Development of preference for the higher probability of reinforcement. Journal of the Experimental Analysis of Behavior.

[46] Trujano RE, López P, Rojas-Leguizamón M, Orduña V (2016). Optimal behavior by rats in a choice task is associated to a persistent conditioned inhibition effect. Behavioural Processes.

[47] Hursh SR, Silberberg A (2008). Economic demand and essential value. Psychological review.

[48] Bickel WK, Marsch LA, Carroll ME (2000). Deconstructing relative reinforcing efficacy and situating the measures of pharmacological reinforcement with behavioral economics: a theoretical proposal. Psychopharmacology.

[49] Orsini, C. A., Moorman, D. E., Young, J. W., Setlow, B. & Floresco, S. B. Neural mechanisms regulating different forms of risk-related decision-making: Insights from animal models. *Neuroscience & Biobehavioral Reviews* **58**, 147–167, doi:http://dx.doi.org/10.1016/j.neubiorev.2015.04.009 (2015). 10.1016/j.neubiorev.2015.04.009 PMC7913606 26072028

[50] Mazur JE (1997). Choice, delay, probability, and conditioned reinforcement. Animal Learning & Behavior.

[51] Dunn R, Spetch ML (1990). Choice with uncertain outcomes: Conditioned reinforcement effects. Journal of the Experimental Analysis of Behavior.

[52] Chow, J. J., Smith, A. P., Wilson, A. G., Zentall, T. R. & Beckmann, J. S. Suboptimal choice in rats: Incentive salience attribution promotes maladaptive decision-making. *Behavioural Brain Research* **320**, 244–254, doi:http://dx.doi.org/10.1016/j.bbr.2016.12.013 (2017). 10.1016/j.bbr.2016.12.013 PMC5241164 27993692

[53] Blanchard TC, Hayden BY, Bromberg-Martin ES (2015). Orbitofrontal cortex uses distinct codes for different choice attributes in decisions motivated by curiosity. Neuron.

[54] Bromberg-Martin ES, Hikosaka O (2009). Midbrain dopamine neurons signal preference for advance information about upcoming rewards. Neuron.

[55] Meyer G (2004). Neuroendocrine response to casino gambling in problem gamblers. Psychoneuroendocrinology.

[56] Potenza MN (2003). Gambling urges in pathological gambling: a functional magnetic resonance imaging study. Archives of general psychiatry.

[57] Crockford DN, Goodyear B, Edwards J, Quickfall J, el-Guebaly N (2005). Cue-induced brain activity in pathological gamblers. Biological psychiatry.

[58] Goudriaan AE, De Ruiter MB, Van Den Brink W, Oosterlaan J, Veltman DJ (2010). Brain activation patterns associated with cue reactivity and craving in abstinent problem gamblers, heavy smokers and healthy controls: an fMRI study. Addiction biology.

[59] Iigaya K, Story GW, Kurth-Nelson Z, Dolan RJ, Dayan P (2016). The modulation of savouring by prediction error and its effects on choice. Elife.

[60] Potenza MN (2008). The neurobiology of pathological gambling and drug addiction: an overview and new findings. Philosophical Transactions of the Royal Society of London B: Biological Sciences.

[61] van Holst RJ, van den Brink W, Veltman DJ, Goudriaan AE (2010). Why gamblers fail to win: A review of cognitive and neuroimaging findings in pathological gambling. Neuroscience and Biobehavioral Reviews.

[62] van Holst RJ, Veltman DJ, Büchel C, van den Brink W, Goudriaan AE (2012). Distorted expectancy coding in problem gambling: is the addictive in the anticipation?. Biological psychiatry.

[63] Platt ML, Huettel SA (2008). Risky business: the neuroeconomics of decision making under uncertainty. Nature neuroscience.

[64] Pinheiro, J., Bates, D., DebRoy, S. & Team, R. C. *nlme*: *Linear and nonlinear mixed effects models*. R package version 3.1–128 (2016).

[65] Wagenmakers E-J, Farrell S (2004). AIC model selection using Akaike weights. Psychon Bull Rev.

