# Supplementary material for: Gambling-like behavior in pigeons: ‘jackpot’ signals promote maladaptive risky choice
Source: Sci Rep. 2017 Jul 26;7:6625. doi: 10.1038/s41598-017-06641-x (PMC5529572; doi:10.1038/s41598-017-06641-x)
Supplement: Supplementary file 1 — Supplementary Information [file 41598_2017_6641_MOESM1_ESM.pdf]

Gambling-like behavior in pigeons: ‘jackpot’ signals promote maladaptive risky choice

Supplementary Materials

Aaron P. Smith, Joshua S. Beckmann, & Thomas R. Zentall

Additional Methods

Reversal. Subjects were again trained on the magnitude discrimination procedure except that the contingencies associated with the two alternatives were reversed: the previous UL alternative was now the CS alternative and vice versa. Training continued until all subjects chose the UL alternative 95% of the time for two consecutive sessions.

After retraining the magnitude discrimination, subjects again trained on the PD procedure except that pigeons previously in the Signaled group were now in the Unsignaled group and pigeons in the Unsignaled group were in the Signaled group. Training continued until the same criterion was met in Experiment 1, lasting 30 sessions for all subjects.

Signaled Losses. All contingencies remained the same as the reversal phase except a novel terminal link stimulus (blue or yellow) now preceded loss outcomes following UL choices for the Signaled group for 10 s followed by the ITI. Training lasted for 10 sessions for all subjects.

Increasing the Cost of Gambling. Experiment 3 began immediately after Experiment 2b. All contingencies (including the addition of the signaled losses) remained the same except that the response requirement to choose the UL alternative for both groups was systematically increased from a fixed ratio (FR) 1 to an FR 2, 4, 8, and 16 across blocks of sessions. The increased response requirement occurred on both forced and free choice trials. Training at each FR continued for 10 sessions as determined by nlme analysis showing no changes in Equation 1 parameters between the average of the first and last five training sessions for both groups.

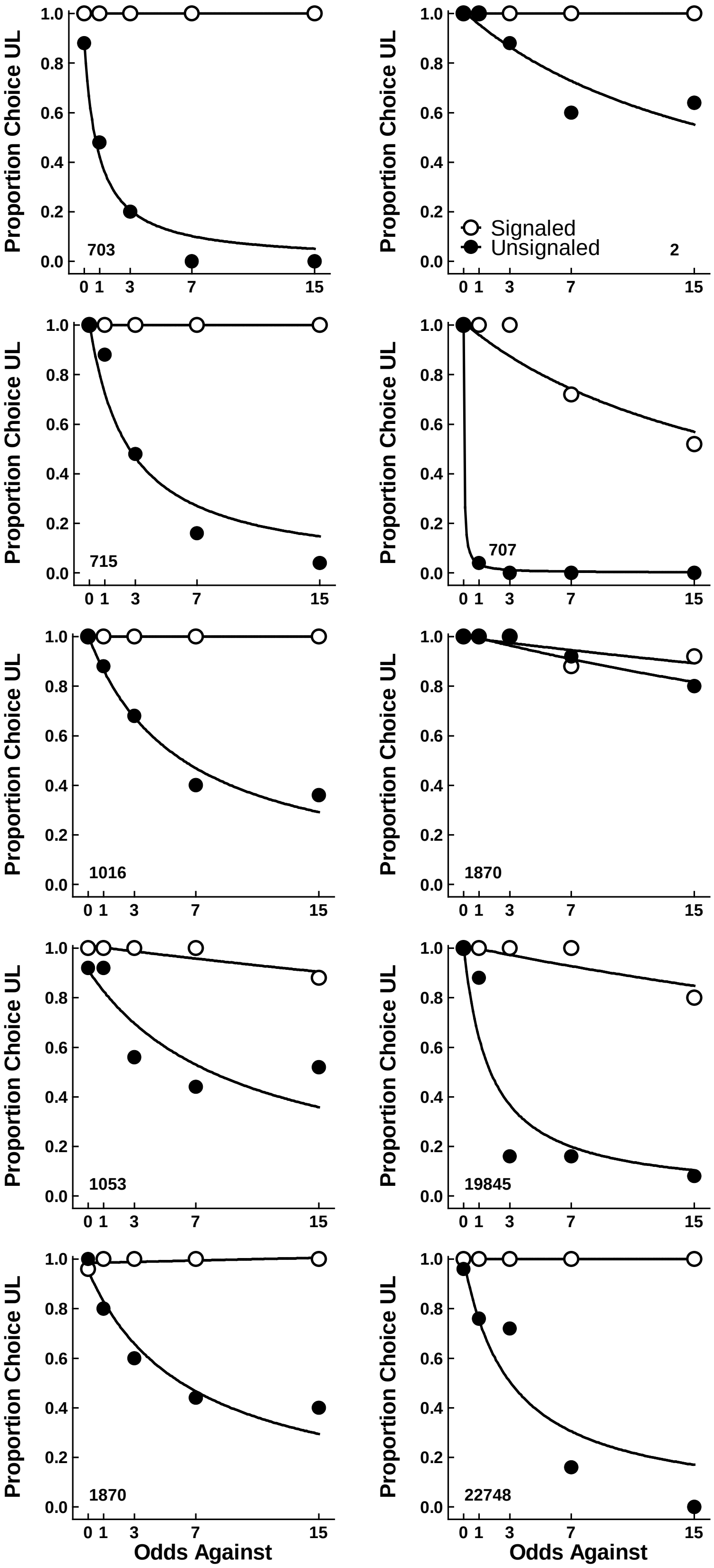

Proportion Choice UL
Odds Against
Signaled
Unsignaled
703
2
715
707
1016
1870
1053
19845
1870
22748

Supplementary Figure S1. Mean proportion choice of the UL alternative for the signaled and unsignaled conditions for each individual as a function of the odds against receiving its reward. The left column shows the pigeons who trained with the signaled condition first, while the right column shows the pigeons who trained with the unsignaled condition first.

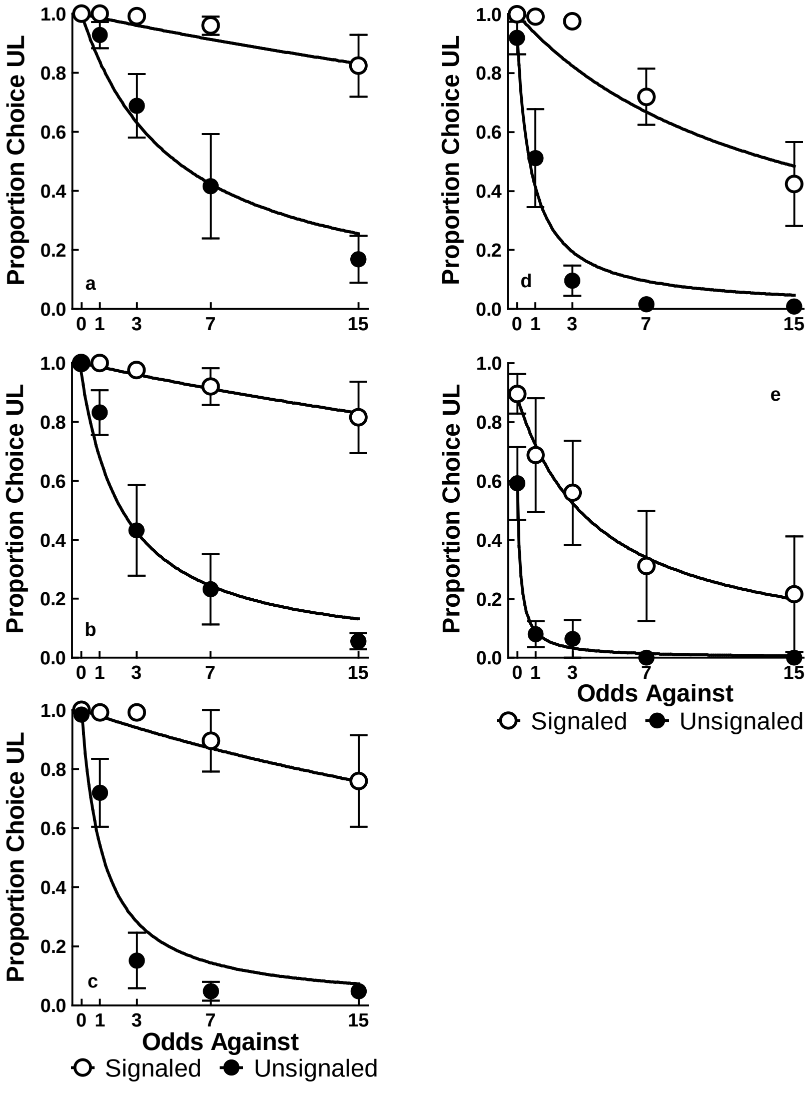

Supplementary Figure S2. Mean (±SEM) proportion choice of the UL alternative for the Signaled and Unsignaled groups as a function of the odds against receiving its reward at each UL peck requirement of 1 (**A**), 2 (**B**), 4 (**C**), 8 (**D**), and 16 (**E**).

Stimulus Value and the Contextual Choice Model

The Equation 1 discounting function is useful in characterizing differences between individuals and groups, but it does not clarify potential mechanisms that underlie these differences. Alternatively, the contextual choice model (CCM) [1], a temporally constrained derivation of the concatenated matching law, can be applied to suboptimal choice data [2]. Because parameters such as the delays for initial and terminal links (the temporal context) were not manipulated, the CCM simplifies to the concatenated generalized matching supplementary equation [3] and takes the following form:

$$\frac{UL}{UL+CS} = \frac{1}{1+\left(\frac{P_{UL}}{P_{CS}}\right)^{S_P} * \left(\frac{M_{UL}}{M_{CS}}\right)^{S_M}} \quad (1)$$

where the proportion UL choice is a function of an individual's sensitivities, S_P and S_M, to the discriminable dimensions of the procedure: probability, *P*, and magnitude, *M*. Using Supplementary Equation 1, group and individual differences can be characterized as due to different sensitivities to the dimensions of reinforcement.

We fit Supplementary Equation 1 to the data at baseline and the reversal using nonlinear regression and constrained the parameters between 0 and -6 with more negative values indicating greater sensitivity. Shown in Supplementary Figure S3, it can be seen that pigeons in the signaled conditions at baseline, S_P = -1.63, S_M = -6, and the reversal, S_P = -1.53, S_M = -4.28, showed greater sensitivities to magnitude than probability relative to the unsignaled conditions at baseline, S_P = -1.15, S_M = -1.44, and the reversal, S_P = -1.36, S_M = -1.54. While using the discounting Equation 1 indicated suboptimal choice as due to a lack of discounting (or UL devaluation), Supplementary Equation 1 characterizes it in terms of a heightened sensitivity to the magnitude of the UL reward. As the scheduled magnitude for UL win trials was 4 pellets for

the signaled and unsignaled conditions, though, it can be inferred that the 'jackpot' signals in the signaled condition effectively further increased the UL magnitude of reinforcement.

To accommodate the price manipulation of increasing the FR requirement to choose the UL, the ratio of FRs between UL/CS can be entered into the model and raised to a sensitivity parameter. A model taking the form of Equation 1 including the new FR ratio (AIC = -71.77) was tested against Equation 1 by itself (AIC = 78.02) for all choice functions at each FR in one model, revealing that including the FR ratio made a substantial improvement to the fit (Δ = -149.79) and that the stimulus value hypothesis could account for these data.

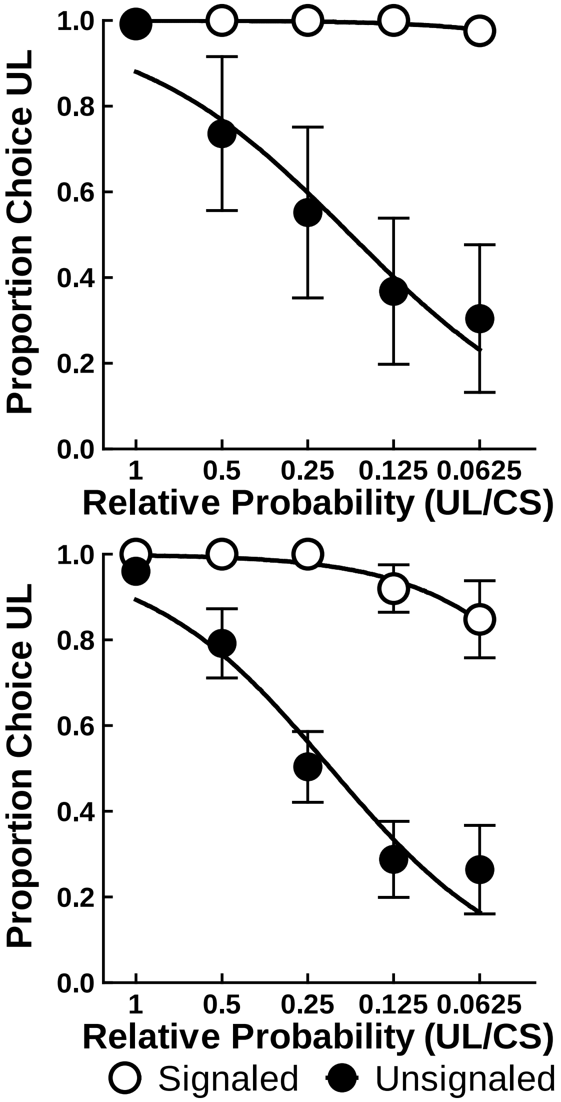

Supplementary Figure S3. Mean (± SEM) proportion choice of the UL alternative averaged over the last five sessions of baseline training (top; n = 10) and the reversal (bottom; n = 10) fit with Equation 3. *Note: the x-axis has been logged for visualization purposes*.

Supplementary References

1 Grace, R. C. A contextual model of concurrent-chains choice. *Journal of the Experimental Analysis of Behavior* **61**, 113-129 (1994).

2 Smith, A. P., Bailey, A. R., Chow, J. J., Beckmann, J. S. & Zentall, T. R. Suboptimal choice in pigeons: Stimulus value predicts choice over frequencies. *PloS one* **11**, e0159336 (2016).

3 Baum, W. M. On two types of deviation from the matching law: bias and undermatching. *Journal of the experimental analysis of behavior* **22**, 231-242 (1974).
